# Applying 3D Models of Giant Salamanders to Explore Form–Function Relationships in Early Digit-Bearing Tetrapods

**DOI:** 10.1093/icb/icae129

**Published:** 2024-08-02

**Authors:** Sandy M Kawano, Johnson Martin, Joshua Medina, Conor Doherty, Gary Zheng, Emma Hsiao, Matthew J Evans, Kevin de Queiroz, R Alexander Pyron, Jonathan M Huie, Riley Lima, Esther M Langan, Alan Peters, Duncan J Irschick

**Affiliations:** Department of Biological Sciences, The George Washington University, 2029 G Street NW, Washington, DC 20052, USA; https://johnsonlm.com, Wilmore, KY 40390, USA; Department of Biology, University of Massachusetts at Amherst, Amherst, MA 01003, USA; Department of Biology, University of Massachusetts at Amherst, Amherst, MA 01003, USA; Department of Biology, University of Massachusetts at Amherst, Amherst, MA 01003, USA; Department of Biology, University of Massachusetts at Amherst, Amherst, MA 01003, USA; Smithsonian National Zoo Conservation Biology Institute, 3001 Connecticut Avenue NW, Washington, DC 20008, USA; Division of Amphibians and Reptiles, National Museum of Natural History, 10th Street & Constitution Avenue NW, Washington, DC 20560, USA; Department of Biological Sciences, The George Washington University, 2029 G Street NW, Washington, DC 20052, USA; Division of Amphibians and Reptiles, National Museum of Natural History, 10th Street & Constitution Avenue NW, Washington, DC 20560, USA; Department of Biological Sciences, The George Washington University, 2029 G Street NW, Washington, DC 20052, USA; Department of Biological Sciences, The George Washington University, 2029 G Street NW, Washington, DC 20052, USA; Division of Amphibians and Reptiles, National Museum of Natural History, 10th Street & Constitution Avenue NW, Washington, DC 20560, USA; Smithsonian National Zoo Conservation Biology Institute, 3001 Connecticut Avenue NW, Washington, DC 20008, USA; Department of Biology, University of Massachusetts at Amherst, Amherst, MA 01003, USA

## Abstract

Extant salamanders are used as modern analogs of early digit-bearing tetrapods due to general similarities in morphology and ecology, but the study species have been primarily terrestrial and relatively smaller when the earliest digit-bearing tetrapods were aquatic and an order of magnitude larger. Thus, we created a 3D computational model of underwater walking in extant Japanese giant salamanders (*Andrias japonicus)* using 3D photogrammetry and open-access graphics software (Blender) to broaden the range of testable hypotheses about the incipient stages of terrestrial locomotion. Our 3D model and software protocol represent the initial stages of an open-access pipeline that could serve as a “one-stop-shop” for studying locomotor function, from creating 3D models to analyzing the mechanics of locomotor gaits. While other pipelines generally require multiple software programs to accomplish the different steps in creating and analyzing computational models of locomotion, our protocol is built entirely within Blender and fully customizable with its Python scripting so users can devote more time to creating and analyzing models instead of navigating the learning curves of several software programs. The main value of our approach is that key kinematic variables (e.g. speed, stride length, and elbow flexion) can be easily altered on the 3D model, allowing scientists to test hypotheses about locomotor function and conduct manipulative experiments (e.g. lengthening bones) that are difficult to perform *in vivo*. The accurate 3D meshes (and animations) generated through photogrammetry also provide exciting opportunities to expand the abundance and diversity of 3D digital animals available for researchers, educators, artists, conservation biologists, etc. to maximize societal impacts.

## Introduction

### Computational models of locomotion

Recent software advances in 3D imaging and modeling provide new opportunities for scientists to understand complex functional processes and patterns of body shape through the lens of 3D computer simulations (Liu 2002; Dong et al. 2010; Laforsch et al. 2012; Demuth et al. 2023). For example, new applications of technologies for modeling living animals, such as 3D photogrammetry (Falkingham 2012; Irschick et al. 2020a, 2020b; Irschick et al. 2022), allow users to examine the functional consequences of morphological variation, as well as potentially develop new metrics for understanding locomotion and robotics. Biomechanical models are particularly important in this context, because general principles from engineering can be applied to biological systems to design and manipulate the mechanical function and performance of structures, as well as understand the physical forces that affect the movement of these structures (Biewener 2003; Irschick and Higham 2016; Biewener and Patek 2018). Conceptual, physical, and mathematical models have commonly been used in biomechanics to identify the mechanistic links between form and function (Alexander 2003), enabling significant contributions about motor control to both the basic and applied sciences.

Computational techniques to study neuromuscular control were crucial to making key scientific discoveries, such as determining that *Tyrannosaurus rex* lacked the muscle anatomy to be a fast runner (Hutchinson and Garcia 2002; Hutchinson et al. 2005). In addition, computational models revealed that chimpanzee muscles do not actually have “supernatural” strength compared to other primates; instead, humans are the anomaly by evolving weaker muscles that are better built for endurance rather than strength (O'Neill et al. 2017). Various software applications can be used to construct musculoskeletal models and then conduct dynamic simulations of motion to identify the mechanical requirements of different locomotor behaviors. For example, OpenSim enables users to biomechanically model the locomotor functions of different musculoskeletal designs, conduct manipulative experiments via computer simulations, and apply algorithms to solve analytical problems in the neuromuscular control of movement (Delp et al. 2007). Multi-joint dynamics with Contact (“MuJoCo”) is a physics engine that has grown in popularity because it can accomplish similar tasks as OpenSim but at a rate that is 600 times faster due to the use of machine learning (Todorov et al. 2012; Richards et al. 2018; La Barbera et al. 2022). Although most physical models used in such simulations have traditionally focused on mammals (e.g. humans, non-human primates, and rodents), valuable information about locomotor evolution has been gleaned from studying a broader range of taxa, such as dinosaurs, frogs, and early digit-bearing tetrapods as well as their modern analogs (Hutchinson et al. 2005; Pierce et al. 2012; Richards et al. 2018; Bishop et al. 2021; Molnar et al. 2021).

Two general goals are, therefore, to broaden the range of accurate 3D models for non-model species, and then to provide a framework to use these 3D models to examine the mechanistic links between morphological and functional diversity in locomotor systems. The latter is especially important, as studies of sprawling crown tetrapods (e.g. lizards, salamanders) are typically limited to high-speed videography, yet debates about the kinds of movements (kinematics) these animals undertake (e.g. pelvic rotation, femur rotation, and femur retraction; see Reilly and Delancey 1997; Reilly 1998; Jayne and Irschick 1999) require a greater range of data than is typically achievable with standard laboratory techniques (i.e. surface landmarks). This is because certain movements are infrequently observed (e.g. sprawling walk without lateral bending) or challenging to quantify without x-ray video (e.g. degree of long-axis rotation in the femur). Creating a 3D model of a sprawling digit-bearing tetrapod in which certain axial and appendicular movements can be manipulated in an experimental manner would, therefore, be a valuable contribution to science by expanding the range of hypotheses that could be tested about locomotor function.

To this end, we created an accurate 3D model of a large crown salamander (Japanese giant salamander, *Andrias japonicus*), and then used that model in a new pipeline we created in open-access, open-source software (Blender) to explore locomotor principles through the visualization and manipulation of kinematic variables. In this way, we set the stage for creating 3D digital models of living animals from 2D photographs, or through other 3D imaging techniques, and then studying dynamics in the locomotor mechanics of these digital models. We then discuss the value of this 3D model for understanding the evolution of terrestrial locomotion in vertebrates, as this and other salamanders are used as modern analogs of early digit-bearing tetrapods due to general similarities in morphology and ecology (Karakasiliotis et al. 2013; Bishop et al. 2015). We first provide some background on salamanders as a model system for studying the locomotion of early digit-bearing tetrapods, and then describe the benefits of integrating computational models into classrooms to improve equity, inclusion, and accessibility in Science, Technology, Engineering, and Mathematics (STEM).

### Use-case: modeling early digit-bearing tetrapods

**Fig. 1:**
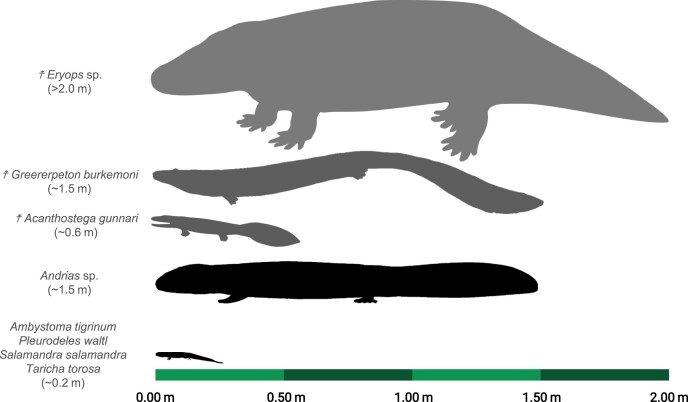
Comparison of body sizes across extant salamanders and stem tetrapods^✝^. Most studies on extant salamanders have been conducted on species that are only about 13–33% of the total length of early digit-bearing stem tetrapods, such as *Greererpeton* and *Acanthostega*, respectively. PhyloPic images were used for *Ambystoma tigrinum* via an Attribution 3.0 Unported license (Quigley 2021), *Acanthostega gunnari* via a CC0 1.0 Universal Public Domain Dedication (Hartman 2013), and *Eryops megacephalus* via an Attribution-NonCommercial-ShareAlike 3.0 Unported license (McHugh 2014). The image for *Greererpeton* was originally created by Gabriel N. Ugueto (Ugueto 2018) and then modified into a gray silhouette, with permission from Gabriel N. Ugueto to use the modified image herein.

Terrestrial locomotion was a major evolutionary transition that led to the expansion of vertebrates into new ecological niches and the subsequent diversification of digit-bearing tetrapods (Fig. 1). Although the fin-to-limb transition was once presumed to have coincided with the water-to-land transition, the earliest digit-bearing tetrapods were fully aquatic and likely used their limbs for underwater walking (Lebedev 1997; Pierce et al. 2013). Extant salamanders represent modern analogs for early digit-bearing tetrapods due to morphological similarities that remained relatively conserved for at least 230 million years (Schoch et al. 2020) and similarities in developmental strategies (Schoch 2002; Long and Gordon 2004). However, extant salamanders commonly studied in this context [e.g. *Ambystoma* (Kawano et al. 2016), *Salamandra* (Pierce et al. 2020), and *Dicamptodon* (Ashley-Ross 1994a)] are highly terrestrial (following ecological classifications from Fabre et al. 2020) and, therefore, more appropriate for modeling more crownward stem tetrapods (Pierce et al. 2013).

The current literature on the locomotor biomechanics of salamanders may reflect a relatively biased sampling of small, terrestrial species that underestimates the functional diversity of salamanders. The neuromuscular control of walking is proposed to be relatively conserved in crown tetrapods (Ashley-Ross 1995; Jayne and Irschick 1999; Reilly et al. 2006; Pierce et al. 2020)—even across aquatic and terrestrial environments (Ashley-Ross and Bechtel 2004)—but broader comparisons across extant salamander species suggest otherwise. Compared to terrestrial salamanders (*A. tigrinum*), the limbs of semi-aquatic salamanders (*Pleurodeles waltl*) support less weight and transmit locomotor forces (i.e. ground reaction forces) more horizontally rather than front-to-back during terrestrial walking, presumably to prioritize stability over forward propulsion (Kawano and Blob 2022). In addition, the mechanics of salamander walking has been limited to species that are about 0.2 m in total length but early digit-bearing tetrapods were an order of magnitude larger (Fig. 1, Godfrey 1989; Clack 2002). Scaling can drastically affect the functional morphology and biomechanics of tetrapod locomotion, such as increasing peak stresses applied to limb bones during terrestrial locomotion (Biewener 2005). Body size likely constrained terrestrial locomotion in early digit-bearing tetrapods, but appropriately sized models are needed to investigate this further.

Japanese giant salamanders—*A. japonicus* (Temminck 1836)—are the second largest amphibians on earth, reaching total lengths of ∼1.5 m and body masses up to 35 kg (Raffaëlli 2022) that are comparable to the estimated body sizes of some early digit-bearing tetrapods. *Greererpeton* closely resembles the morphology and ecology of *Andrias*, which includes dorsoventrally compressed bodies that are characteristic of benthic walkers, a well-developed lateral line system, relatively short limbs, and a primarily aquatic lifestyle (Godfrey 1989) with the potential for limited excursions on land (Whitney and Pierce 2021). *Andrias* inhabit fast-flowing streams and rivers that are primarily in southern Honshū, Shikoku, and Kyūshū islands in Japan. Although they are almost entirely aquatic, they engage in extensive walking on riverbeds and can climb up artificial inclines and step ladders (Takahashi et al. 2016). However, live *Andrias* are difficult to study due to their restricted geographic ranges and rarity (listed as vulnerable in the 2021 IUCN reclassification: IUCN SSC Amphibian Specialist Group 2022), so little locomotor work has been done on this species. To fill this gap, we created a 3D model of an adult *A. japonicus*, which should allow for more hypothesis testing related to sprawling locomotion in quadrupedal vertebrates, such as early digit-bearing tetrapods. Ultimately, we aim to develop a user-friendly pipeline that could be a central hub for creating, animating, and analyzing animal movements.

## Methodology

### Creating a 3D model

**Fig. 2:**
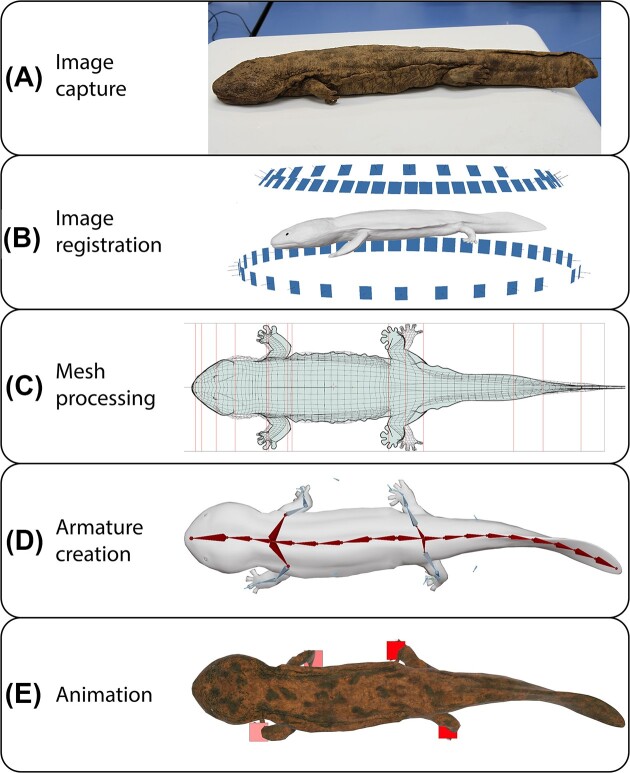
Overview of our workflow to generate and animate 3D photogrammetry models. **(A)** The *A. japonicus* specimen is photographed from a successive series of angles around the specimen. **(B)** Through digital photogrammetry, the 3D meshes from the dorsal and ventral views are reconstructed from the specimen photos. Relative photo positions are illustrated as blue rectangles, with each photo contributing to the central 3D mesh (dorsal mesh pictured here). **(C)** The mesh is processed using 3D modeling software. Dorsal and ventral meshes are combined, with the resulting wireframe visible in black. Additional specimen measurements, visible as red lines and a blue silhouette, are used to correct for artifacts in the scan data. **(D)** A 3D animation of the armature is created as a series of interconnected bones, controlled via inverse-kinematic constraints on the “bones,” depicted as primitive geometries. **(E)** The armature is animated using reference videos and known kinematic data. A texture map is generated using specimen photos. Virtual trackers attached to the feet keep track of stride length throughout the animation.

To create a 3D model of an adult Japanese giant salamander, we integrated data from a 3D digital photogrammetry scan of a preserved individual with video data from live individuals. Photos of underwater walking and the general morphology of live *A. japonicus* were obtained from the Reptile Discovery Center (RDC) at the Smithsonian National Zoo Conservation Biology Institute (NZCBI) through a protocol approved by their Animal Care and Use Committee (protocol number: SI-22,035). The two individuals tested in July 2022 were two large males: “Hiro” (RDC accession number: 307,252; 87 cm total length and 7.8 kg total weight) and an individual in the public exhibit (RDC accession number: 307,247; 91 cm total length and 7.1 kg total weight), which were donated along with two females by the Asa Zoological Park in the Asakita Ward of Hiroshima, Japan. We filmed the two males at different angles with a GoPro HERO10 Black camera. The camera was submerged underwater, or positioned outside of the enclosure to obtain narrow and wide fields of view, respectively. The camera and camera accessories were disinfected prior to use in the animal enclosures. The videos were then decomposed into consecutive series of photographs for the next step of the workflow (i.e. image registration). Further, we followed general guidelines for single-camera photogrammetry to convert 2D images into 3D models (Falkingham 2012; Medina et al. 2020; Fig. 2).

Preserved specimens were obtained from the National Museum of Natural History (NMNH) in Washington, D.C., to capture finer resolution of skin texture and general body proportions for our 3D model (Fig. 2A). We chose two of the largest specimens in the collection, USNM 34215 [total length: 64 cm, snout vent length (SVL): 43 cm] and USNM 56769 (total length: 66 cm, SVL 43 cm). The specimens were stored in ethanol and carefully dried with paper towels to reduce image distortion that can occur from light refracting through or reflecting from wet surfaces. We used a plastic dissection tray as a turntable to manually rotate the specimen 360° while the camera was stationary. The turntable was rotated only a few degrees between each photograph to create smooth transitions.

Each specimen was photographed using a Canon EF 24–105 mm f/4L IS USM lens fitted with a UV non-polarizing filter on a Canon EOS 7D digital camera. The camera was mounted onto a Manfrotto aluminum tripod with a three-way head (model: MK055XPRO3-3W) to improve image stabilization. A consecutive series of photographs was taken 360° around the specimen at a constant angle (pointed downwards at roughly 45° above the specimen) and then repeated at another constant angle (roughly lateral to the specimen). These photographs were collected while the specimen was oriented ventral-side down and then the process was repeated on the opposite side to capture enough detail around the body. 2D photos were stitched together to form a 3D model by aligning common landmarks across photos through image registration (Fig. 2B), which was facilitated by incorporating 50% or more overlap between consecutive photos and photographing specimens from at least two camera angles during image capture (Medina et al. 2020). In addition, image parameters were made consistent across photos to account for differences in image quality, lighting conditions, and camera settings between the photo shoots at the NZCBI and NMNH. In the present paper, we describe the workflow for the 3D model created for USNM 34,215.

Separate 3D models were created for each anatomical perspective (e.g. dorsal vs. ventral) using standard techniques for photogrammetry reconstruction (Bot and Irschick 2019) and then merged into a single 3D model file using Blender (version 3.4). A dorsal mesh was reconstructed using 41 images of the specimen and a ventral mesh was constructed from a different set of 47 images of the same specimen. The combined 3D mesh was constructed using polygon modeling techniques (see Bot and Irschick 2019 for more detail) overlaid on top of the two separate 3D models, matching the shape and size of the latter. Certain areas of the 3D model were corrected using 2D images created in Medibang Paint Pro as well as measurements that were referenced from USNM 56769 and living specimens at the NZCBI to account for minor artifacts created during the photogrammetry process (Fig. 2C).

The combined 3D model was also optimized for broader animation uses by applying polygon modeling and retopology methods (refer to Bot and Irschick 2019 for more details) that allow the animation to be modified on computer workstations with a wide range of computing power. The 3D model and subsequent animations, simulations, and measurements were created on a Windows 11 workstation with a 6-Core Intel i7-8700 processor, NVIDIA Geforce 1070 graphics card, and 64 gigabytes of RAM (random access memory). Additional measurements and modifications were also performed on an Apple M1 processor and a variety of other Intel workstations to confirm functionality across a variety of operating platforms and hardware specifications. As detailed in prior publications (Falkingham 2012; Irschick et al. 2020a, 2020b; Medina et al. 2020; Irschick et al. 2022), there is a long and established empirical history establishing 3D photogrammetry as being accurate, as long as certain conditions are met (Zhang and Maga 2023), which we have generally adhered to here. The accuracy of our combined model later assisted in improving the development of the 3D armature (“digital marionette”) and subsequent animations and simulations.

### Animating the 3D model

An animated 3D armature was built and attached to the combined 3D model to form the digital model used for simulation and measurements (Fig. 2D). The “skeleton” of the armature was developed based on the general anatomy of the specimens while maintaining a level of simplicity to improve the usability of the armature by users with a wide range of technical skills. The armature consisted of a series of connected rigid geometries (“bones”) that were attached to the mesh of the 3D model using vertex weights, which were applied based on the anatomy of the specimen and the proximity of each bone to each vertex of the mesh. In Blender, bones are simplified geometries to facilitate rigging and animating the armature rather than the actual bones of the specimen. The connected bones were then modified with controllers using inverse-kinematic bone constraints, allowing one to reconstruct the kinematics (“motions”) that would position an articulated skeleton into a target pose (e.g. foot in contact with ground).

The resulting armature allowed more realistic control of the bones through Blender's posing tools than a basic forward-kinematic model would have enabled. Additionally, the 3D mesh connected to and surrounding the 3D armature allowed for better detection of the plausible rotations of each joint by giving the user feedback on where limb and body intersections may occur when certain rational values were inputted (i.e. to avoid collisions between ipsilateral forelimbs and hindlimbs). At this stage, the 3D mesh and armature were scaled to reflect the total length of specimen USNM 34215 (64 cm). Afterwards, we scaled the model to reflect the total length of an early digit-bearing tetrapod (total length: 1.2 m, SVL: 0.8 m). The coordinate system was set to a standard XYZ Euler rotation mode. The static 3D mesh is available on SketchFab for general use (https://skfb.ly/oIWro) and version controlled in our GitHub repository (https://github.com/JohnsonLM/blender-tetrapod-toolkit/).

The digitally reconstructed mesh (Fig. 2) was initially animated using reference videos collected from live *A. japonicus* at the NZCBI and then modified as needed with published values of walking kinematics in *Dicamptodon tenebrosus* and *Taricha torosa*. These species were chosen because *Dicamptodon* is made up of some of the largest salamanders studied whose kinematic data are available and *T. torosa* is one of the few species of semi-aquatic salamanders whose kinematics have been quantified during underwater and terrestrial walking (Ashley-Ross 1994b, 1994a; Ashley-Ross and Bechtel 2004). We could not rely solely on kinematic data from the GoPro videos of the NZCBI's live *A. japonicus* because environmental enrichment in their enclosures prevented us from recording consistently straight walking bouts. While there are likely to be differences between these species, it provides a reasonable starting point of comparison to ground-truth our data. For instance, our *Andrias* model exhibits similar gait patterns as *Dicamptodon* and *Taricha*. Limbs within a diagonal couplet (e.g. left forelimb and right hindlimb) were programmed to move roughly in phase with each other in our *Andrias* model and out of phase with the opposite diagonal couplet to produce a trot-like walk with a duty factor of ∼52%, which is comparable to 56–57% during terrestrial walking in *D. tenebrosus* and intermediate between the 41% during underwater walking and 77% during terrestrial walking of *T. torosa* (Bennett et al. 2001; Ashley-Ross et al. 2009).

We also verified that our *Andrias* model had a realistic stride length and stride frequency. Small virtual trackers were placed onto the armature bones in the model's feet and used as reference points to assist in measuring stride length. Stride length was measured by finding the point of the initial footfall of the left hindlimb, marked by the virtual tracker, and then playing the animation until the next footfall. Single measurements of stride length were conducted with the Blender add-on “Measure-It,” which provides more accurate measurements compared to the base “Measure” tool. These tools must be reapplied for each frame of movement, however, so we created a Tetrapod Toolkit in Blender that can be used to automatically measure length and rotational changes of bones across multiple frames. Stride frequency was calculated as the inverse of the number of frames per stride multiplied by the frame rate of the animation (30 frames/s).

The stride length of our digital *Andrias* model was comparable to other slow-moving salamanders. The relative stride length is the stride length divided by SVL, and was 39.5% SVL in our model. Similarly, the relative stride length in slow-moving individuals was 47% in California tiger salamanders *(Ambystoma californiense*), 54% in fire salamanders (*Salamandra salamandra*), and as low as 20% in some plethodontids, although the latter includes some elongate species (Edwards 1977). The relative stride lengths of larval and metamorphosed *Dicamptodon* were 64.7 ± 6.7% and 65.6 ± 4.8% SVL, respectively, while they were walking at less than 0.5 SVL/s with stride frequencies of ∼0.57 strides/s and ∼0.66 strides/s, respectively (Ashley-Ross 1994b). *Andrias japonicus* has relatively short limbs so it is reasonable to expect its relative stride length to be shorter than species with relatively longer limbs. The stride frequency of our *Andrias* model was 0.353 strides/s which is almost 50% slower than *Dicamptodon*, matching general principles that larger animals tend to have slower stride frequencies than smaller animals (Heglund and Taylor 1988).

Sprawling postures in digit-bearing tetrapods are characterized by a diagonal couplet gait that are often also accompanied by lateral bending of the trunk (Ashley-Ross 1994a). The animation of each bone in the armature was achieved by applying a function modifier to the X-, Y-, and Z-axes under the rotation channels in Blender to reanimate each joint to approximate the reference data from Ashley-Ross and Bechtel (2004) and Ashley-Ross (1994a). To make it easier for users to measure the rotational and location data along the X-, Y-, and Z-axes of the armature's bones at each frame of movement, we created a Tetrapod Toolkit add-on for Blender that is available on our GitHub repository. The animated model is available on Sketchfab (https://skfb.ly/oIWrn) and version-controlled on our GitHub repository. In all, the total amount of time spent on recreating the salamander, as well as the animations, amounted to over 60 hours of work but it is expected that this would be greatly reduced in future reconstructions, given the knowledge gained from this process.

### Proof-of-concept: how lateral bending affects stride length

Lateral bending of the trunk is commonly observed during terrestrial locomotion in quadrupedal tetrapods, and generally increases organismal speed (Ritter 1992). Organismal speed can be increased by changing their stride (also called “limb cycle”) which is composed of the stance phase (when the foot is in contact with the ground) and swing phase (when the foot is airborne). Stride length is the distance traveled during one stride (i.e. two consecutive footfalls of the same foot), whereas stride frequency is the number of strides observed per second (McElroy and Reilly 2009). Although limb length affects whether stride length or stride frequency is modified to increase organismal speed in lizards (McElroy and Reilly 2009), salamanders generally increase their stride length by increasing lateral bending of their trunk (Ashley-Ross 1994b, 1994a; Ashley-Ross and Bechtel 2004). But, there may be a limit. Excessive lateral bending can increase instability at higher magnitudes which would decrease forward movement (Edwards 1977); however, this can be difficult to observe in live animals without inducing an escape response.

The concept that lateral bending increases stride length has long been proposed but difficult to mechanistically test *in vivo* (Snyder 1952; Ritter 1992; Ashley-Ross 1994a). Turtles are useful to test this hypothesis because their vertebrae are fused to the inner part of their carapace (upper shell) so changes in their locomotor performance must be accomplished with their limbs, either by increasing girdle rotations or by limb protraction (Schmidt et al. 2016; Mayerl et al. 2019; Vega and Ashley-Ross 2021). Recently, the effects of reduced lateral bending on the walking kinematics of tiger salamanders (*A. tigrinum*) were tested by “turtling” individuals with flexible or rigid tubes surrounding their trunk and then comparing their kinematics to a control group of individuals without tubes (Vega and Ashley-Ross 2021). Despite the tubes fully or partially restricting lateral bending in individuals with rigid and flexible tubes, respectively, there were no differences in limb kinematics across the experimental and control groups. Instead, restricting lateral bending of the trunk caused individuals to laterally bend their tails that are normally held straight and dragged during walking (Vega and Ashley-Ross 2021). However, the added mass of the tubes might have affected their locomotor performance since walking speeds were slower in the experimental groups. By testing this hypothesis with our 3D *Andrias* model, users can remove extraneous variables that could affect their results.

**Fig. 3:**
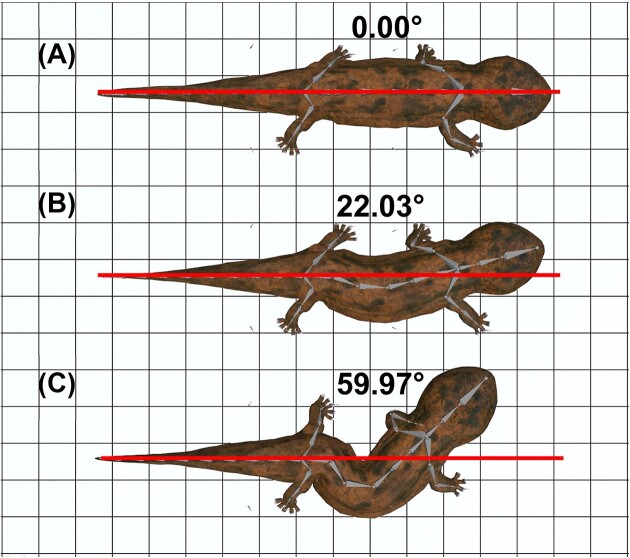
Comparison of different amplitudes of lateral bending in the 3D *Andrias* model. The extent of lateral bending at the trunk can be modified by changing the amplitude of the trunk armature (gray) relative to the reference line (red). We created three models with **(A)** no lateral bending (0°), **(B)** moderate lateral bending (22.03°), and **(C)** extreme lateral bending (59.97°). Each grid represents a 0.10 × 0.10 m square.

To enable users to examine the relationship between lateral bending and stride length in a salamander, we created an online tutorial using our 3D model, Blender file, and Tetrapod Toolkit Add-on that are available on our GitHub repository. The amplitude of lateral bending along the trunk was programmed as a sine function with modifier functions in Blender that was applied to each rotational axis of interest (Fig. 3). The amplitude was then adjusted to generate three models with no lateral bending (0°), moderate lateral bending (22.03°), and extreme lateral bending (59.97°). The model with moderate lateral bending depicts the most biologically realistic reconstruction of a diagonal couplet salamander of the three models, and was based on measurements of the anterior–posterior trunk angle from *Dicamptodon*, which oscillates from –40° to +20° (Ashley-Ross 1994a). We then compared the output from these models to validate that lateral bending increases stride length when moderate, but decreases stride length when excessive.

Our computational analysis of *A. japonicus* validates a couple of locomotor principles about stride length, stride frequency, organismal speed, and lateral bending (Table 1). Compared to the model with no lateral bending, stride length changes by +1.16% with moderate lateral bending and –13.93% with extreme lateral bending. Inverse changes are observed for stride frequency, albeit not proportional to the changes for stride length. Using lateral bending results in the stride frequency changing by –5.89% when moderate and +2.56% when extreme. Measures of organismal speed followed similar patterns as stride length, whereby the model with a moderate amount of lateral bending was faster than the models with no lateral bending or extreme lateral bending by +5.26% and +17.65%, respectively. Playing the animation in our Blender file allows users to visualize the consequences of these differences on locomotor performance over several strides. The models with no lateral bending and moderate lateral bending were initially programmed to move at the same speed, so the difference in organismal speed during our simulation was due to the increased stride length associated with greater lateral bending. The forward speed of the model with extreme lateral bending was initially the same as the other two models but had to be manually reduced because the limbs could no longer keep up with the body, causing the model to transition to undulatory locomotion at faster speeds as has been documented in other studies of salamander locomotion (Evans 1946). Overall, these corroborate expectations that lateral bending tends to increase stride length and organismal speed but only to a limit.

**Table 1:** Relationships between amount of lateral bending, stride length, and stride frequency.

Lateral bending	Stride length (m)	Stride length (% SVL)	Stride frequency (strides/s)	Speed (m/s)
None	0.313	39.13	0.375	0.057
Moderate	0.316	39.50	0.353	0.060
Extreme	0.209	26.13	0.385	0.051

## Discussion

### Expanding computational analyses of locomotion in different environments and taxa

We created a static and an animated 3D model of an adult Japanese giant salamander (Fig. 2) based on preserved specimens from the Smithsonian NMNH and live specimens from the Smithsonian NZCBI. The animation was created using a customizable Python script, which allows users to modulate basic kinematic parameters, such as stride length and stride frequency, and even contribute additional analyses to expand the utility of this pipeline to a broader range of use-cases. With this code and the 3D model, one can evaluate how changing other stride parameters affects locomotor kinematics (e.g. pelvic rotation, long-axis rotation of the femur)—and vice versa—to identify underlying locomotor patterns in a sprawling quadrupedal tetrapod, which will be the focus of a subsequent manuscript by our team.

A reasonable question is how the methods and approach laid out in this paper provide an upgrade over more commonly used techniques such as stick figure simulations. “Simple” models, such as stick figures, are valuable tools in computational biology because they can be broadly applied to a variety of animals, allow users to individually isolate and systematically manipulate parameters, and simplify analyses (Anderson et al. 2020). However, the trade-off is that several assumptions must often be made to reduce complexity that may not be biologically realistic. Simple models may be used as excellent starting points in comparative studies to examine how additional features might affect the outcome of the model. Compared to stick figure models, our approach incorporates an accurate 3D mesh of a live animal that allows users to test more biologically realistic hypotheses. Importantly, most methods in locomotor analysis are visual in nature. We argue that the ability to visualize a lifelike exterior scan of a salamander using different limb and joint movements during different gaits improves the ability of learners to understand locomotion, particularly for novices or members of the general public who often benefit from having computational models that are less abstract and more relatable.

The current iteration of our model is intended to be a starting point, with a myriad of possibilities to construct more complex models to suit the needs of users. As with OpenSim, the pipeline laid out here also allows more intensive modeling of locomotion, such as adding more bones or joints, muscles, or other anatomical features. In the future, one can take accurate and elaborate computed tomography (CT) scans that are readily available on platforms such as Morphosource or Sketchfab and build an armature based on the actual skeletal elements of the animal to evaluate whether the increased complexity sheds new light on its locomotion. We also note that one of the primary benefits to this procedure is the ability to model locomotion on a broader range of animals, as it is now possible to get accurate 3D surface scans of animals (e.g. rhino, https://skfb.ly/6QTJX), and then insert a digital rig that mimics that individual's anatomy. Our pipeline allows users to build 3D photogrammetry models of living animals, providing the opportunity to model animals that are traditionally difficult to gain access to or study in captivity. Further, the growing stability and popularity of Blender as a well-supported, community-driven, and open-access resource speaks to the value of this process and its longevity.

In this regard, our approach is not meant to be a replacement for other software suites for terrestrial locomotion (e.g. OpenSim) or fluid dynamics (e.g. OpenFOAM), but rather an alternative for users who seek a “Jack-of-All-Trades” software that is easier to adapt as their needs change. Other software suites are often restricted to locomotor analyses on *terra firma* so users must switch to other software for computational fluid dynamics (CFD), but Blender has the option to incorporate CFD directly into its environment through customized routines, such as BlenderFOAM (https://github.com/nathanrooy/BlenderFOAM). Blender is known for its 3D rendering capabilities and has been used by biologists to reconstruct the walking gait of an extinct arachnid (Garwood and Dunlop 2014), and estimate the muscle strain and maximum gape of two extant archosaurs (*Alligator mississippiensis, Buteo buteo*) and three extinct archosaurs (*T. rex, Allosaurus fragilis*, and *Erlikosaurus andrewsi*; Lautenschlager 2015). While other software suites already have workflows for simulating motion in humans and a subset of digit-bearing tetrapods, Blender could have a lower barrier to entry that could foster the development of a more diverse collection of digital animals to study patterns of locomotor principles across taxa and environmental conditions.

In addition, Blender is a versatile software that can be scaled up to complex 3D modeling projects by importing custom computer scripts as “add-ons^”^ to increase the capabilities of the software even further, in a similar way that R and Python packages can be imported to increase the range of tools available. One such example is “Myogenerator,” which allows users to interactively overlay 3D models of muscles over bones in order to estimate the physiological cross-sectional area of individual muscles, bite force of a skull, and lines of action for muscle units (Herbstet al. 2022). Blender can create, modify, and animate 3D meshes which enables users to apply retrodeformation techniques that can fix damaged bones before they collect data from the 3D models (Herbstet al. 2022). As discussed in Bot and Irschick (2019), one can import individual 2D images to improve the level of detail for an existing 3D model, which is an important tool for ensuring accuracy. The ability to create and package custom scripts that build upon an already powerful set of versatile tools to create, sculpt, animate, and analyze 3D models with only one software suite offers promising opportunities to explore new horizons in visualizing and studying animal locomotion.

### Testing long-standing hypotheses about the water-to-land transition

The primary value of this work is providing a tool for manipulating basic gait parameters in a large, aquatic tetrapod with a sprawling limb posture to explore form–function relationships in a modern analog for early digit-bearing tetrapods (Fig. 3). Although early digit-bearing tetrapods are proposed to have used a salamander-like walk on land, the application of 3D imaging techniques indicates that this gait was not possible in what is currently the earliest stem tetrapod to use terrestrial locomotion. 3D reconstructions of the Devonian digit-bearing tetrapod, *Ichthyostega*, indicated that it had limited mobility in its shoulders and hips that precluded it from using a lateral sequence, diagonal couplet walk that is typified in sprawling quadrupedal tetrapods (Pierce et al. 2012, 2013). *Ichthyostega* also could not laterally bend its trunk due to ribs with overlapping processes that formed a “rigid armor” around the body cavity (Jarvik 1996; Clack 2005). Moreover, its hindlimbs had limited long-axis rotation about the hips compared to extant tetrapods (e.g. salamander, crocodile, platypus, seal, and otter) that prevented its feet from contacting the substrate and generating propulsion on land. Given these features, *Ichthyostega* likely used synchronous movements of its forelimbs to drag the posterior half of its body which was held straight or flexed dorsoventrally in a “crutching” gait on land that is currently only documented in the terrestrial locomotion of mudskipper fishes (Pierce et al. 2012, 2013).

The discovery that there were at least two gaits that early digit-bearing tetrapods may have used during the initial stages of evolving terrestrial locomotion opens fruitful opportunities to test hypotheses about the kinematic features that were needed for effectively moving on land at a time when the musculoskeletal system of stem tetrapods had evolved in aquatic environments for almost 375 million years. Specifically, three main hypotheses that could be tested using our 3D model of *Andrias* include: (1) limited ability to protract the limbs will decrease stride length, (2) increasing the rotation of the shoulder or hip girdles will increase stride length, and (3) increasing the organismal speed of the animal will increase the contributions of girdle rotations towards propulsion while decreasing contributions from limb retraction and long-axis rotation of the limbs. Future manipulations of our model could possibly test these ideas.

Prior researchers have discussed the relative contributions of femur retraction, long-axis rotation of the femur, and pelvic rotation for propelling locomotion in sprawling tetrapods such as salamanders and lizards (Reilly and Delancey 1997; Jayne and Irschick 1999; Reilly et al. 2006). In terrestrial salamanders, propulsion is supplied 56–62% by femur retraction, 26–28% by long-axis rotation of the femur about the hip, and 10–18% by rotation of the pelvic girdle (hips). As walking speed increases, the contributions of femur retraction and long-axis rotation decrease while girdle rotation increases (Edwards 1977). However, studies on amphibians and lizards have traditionally only focused on the hindlimbs since they are the primary propulsors in these taxa but forelimbs exhibit different locomotor kinematics and can contribute towards propulsion, presumably to offset the counteractive effects of tail drag (Willey et al. 2004; Kawano et al. 2016). Manipulating and quantifying the relative contributions of these variables is tricky, though, as their incidence may vary among species, among environment contexts (e.g. uphill and downhill running), and with speed. The use of 3D models, simulations, and computer animation tools ameliorate some of these issues by enabling users to conduct experiments in controlled environments and test hypotheses about form–function relationships that would be challenging to conduct *in vivo*.

Using our model, one can impose specific locomotor scenarios, such as inducing a large amount of pelvic rotation with no long-axis rotation of the femur to systematically test the contributions of each parameter on the metric of locomotor performance (e.g. stride length and walking speed). Users could also examine whether it is possible for a salamander to effectively move on land with a mudskipper-like crutching gait or restrict girdle rotations to quantify the magnitude to which it reduces walking speed, allowing one to mechanistically test the three main hypotheses described earlier regarding the evolution of terrestrial locomotion in stem tetrapods. By providing an accurate 3D model of a giant salamander that can be manipulated in open-access software, we hope to broaden engagement in the use of 3D computational approaches in research and education.

It is also possible to manipulate the dimensions of musculoskeletal elements to mimic different morphological traits of various species. In this regard, this tool could be used to test the functional consequences of morphological changes in limb bones observed in stem tetrapods spanning the transition from aquatic to terrestrial environments. In addition, soft tissue tends to further restrict the range of motion and could be tested *in silico* by modeling muscles onto the 3D model with Myogenerator or 3D geometries that are based on soft-tissue reconstructions of contrast-enhanced specimens (Pierce et al. 2012; Herbstet al. 2022). Users could use these capabilities to test the functional role of the ventral ridge, which is a prominent feature found on the humeri of many Devonian stem tetrapods to presumably increase area for muscle attachment but then regressed as taxa became more terrestrial. Users could download 3D meshes of the non-digit-bearing stem tetrapod, *Tiktaalik roseae* (https://shubinlab.uchicago.edu/research-2-2/), and then modify the size of the ventral ridge to examine how it would affect muscle attachment and, subsequently, the range of motion about the shoulder joint, all within the same Blender file.

### Addressing grand challenges in organismal biology

*“We must stimulate, encourage, and train toolmakers who provide us with the novel approaches to the study of organisms that will open future horizons.”*—Schwenk et al. (2009)

One of the primary aims of the “Computational and physical models in research and teaching to explore form–function relationships” symposium at the 2024 national meeting of the Society for Integrative and Comparative Biology is to promote the use of model-based approaches across a broader scientific community, which we address by developing resources that are accessible to students and non-specialists. The unification and accessibility of tools, data, and other resources have been rate-limiting steps for not only advancing science (Mykles et al. 2010) but also a major barrier to entry for hobbyists or students. In this vein, our work complements and adds to existing online databases that promote accessibility, inclusion, and equity in science by making 3D data of natural history specimens broadly available and open-access. Large 3D databases such as Morphosource (www.Morphosource.org) and the Digital Life Project (www.digitallife3D.org) provide free 3D models of animals or animal structures (Boyer et al. 2016; Bot and Irschick 2019; Irschick et al. 2022). The value of these online databases can be seen in their usage. The Digital Life Project applies photogrammetry and computer animation to create accurate digital copies of animals, with colors and movements that often cannot be preserved in natural history collections (Bot and Irschick 2019). In the past eight years, the Digital Life Project has generated over 100 3D models that have been downloaded over 197,000 times (with over 8K users) from SketchFab (https://sketchfab.com/DigitalLife3D). Moreover, OpenVertebrate (oVert) (https://www.floridamuseum.ufl.edu/overt/) has generated over 30,000 digital files across more than 13K specimens that have been downloaded over 100K times (Blackburn et al. 2024).

As methods to collect natural history data have become more accessible, the traditional perspective of each specimen as a physical object has been transformed to a more contemporary view of representing a multidimensional database. The “extended specimen” was conceptualized to maximize the potential of specimens in natural history collections by integrating information that spanned multiple scales including “genetic, phenotypic, behavioral, and environmental data, as well as biotic interaction networks and new multimedia components (e.g. 2D and 3D specimen images, *in situ* field images, videos of field conditions)” (Lendemer et al. 2020). Although whole-body CT scans and 3D meshes of *A. japonicus* (Japanese giant salamanders) and *A. davidianus* (Chinese giant salamanders) are available on MorphoSource.org, our 3D model of a Japanese giant salamander adds accurate portrayals of texture, color, and locomotor behavior to provide an “extended specimen” (Lendemer et al. 2020) that can be used for a variety of purposes including research, education, conservation management, and art. By contributing to global initiatives that increase access to digital collections of natural history specimens, we hope to foster innovative uses of our 3D model of *A. japonicus* that maximize their societal impacts.

### Equitable access to educational outcomes

One of the key advantages of digital specimens, such as our 3D Japanese giant salamander, is more equitable access to a wider audience, as travel to museums can be time consuming and expensive. In fact, more than 50% of all downloads on Morphosource.org occurred after the COVID-19 pandemic escalated in March 2020 (Blackburn et al. 2024). Virtual collections of natural history specimens are becoming valuable resources to promote equitable educational outcomes for students by providing experiential learning opportunities for online courses and students who cannot attend classes in person due to caretaking responsibilities or mobility impairments (Sidlauskas et al. 2021), as well as instructors who do not have an Institutional Animal Care and Use Committee (IACUC) at their university that could enable experiments on live vertebrate animals.

Interactive 3D computational models also help students develop transferable skills in interdisciplinary collaboration, independent learning, and metacognitive reasoning that align with the core competencies needed to improve biological literacy in undergraduate education (American Association for the Advancement of Science 2011). One of the silver linings of the COVID-19 pandemic is that the lack of access to wet labs motivated many instructors to develop innovative pedagogical techniques for students to glean transferable skills in computational tools that are highly marketable in industry and the STEM workforce (McDonald et al. 2022). In fact, computer simulations lead to higher gains in students’ conceptual knowledge and procedural knowledge while promoting cognitive dissonance that fostered intellectual growth (Smetana and Bell 2012). Experiential learning and student-centered pedagogy improves student engagement and academic performance (Armbruster et al. 2009), and many medical students report that interactive 3D digital models were more effective at learning anatomy and led to higher test scores compared to static 2D images (Triepels et al. 2020).

## Concluding remarks

Lifelike 3D models represent a powerful tool for better understanding organismal body shape, locomotion, and ecology, and provide new resources for conservation and education. Our open-source and open-access workflow for a 3D Japanese giant salamander enables learners to easily participate in discovery-driven and hypothesis-driven science (Mykles et al. 2010). Our approach also builds an infrastructure that enables interdisciplinary training and collaborative opportunities for a wide-range of disciplines, from artists to paleontologists to education specialists at non-profit organizations. As the 3D models and data generated from our software protocol and other computational workflows continue to grow, we anticipate a surge in interdisciplinary syntheses that provide more comprehensive descriptions of the mechanistic links that determine generalizable principles in organismal biology (Padilla et al. 2014).

## Data Availability

The data underlying this article are available in Sketchfab collections for the Digital Life Project: https://sketchfab.com/DigitalLife3D/. Our 3D models of *A. japonicus* are free for non-profit uses, and available as a static mesh or texturized animation. Our 3D models, Blender add-on, example Blender files, and tutorial are available at https://github.com/JohnsonLM/blender-tetrapod-toolkit. Raw copies of digital images, and other digital files used to create the final 3D models will be uploaded to our GitHub repository.

## Notes

From the symposium “Computational and Physical Models in Research and Teaching to Explore Form-Function Relationships” presented at the annual meeting of the Society for Integrative and Comparative Biology, January 2 - 6, 2024.
